# Comparison of Oncologic Outcomes in Laparoscopic versus Open Surgery for Non-Metastatic Colorectal Cancer: Personal Experience in a Single Institution

**DOI:** 10.3390/jcm8060875

**Published:** 2019-06-19

**Authors:** Chong-Chi Chiu, Wen-Li Lin, Hon-Yi Shi, Chien-Cheng Huang, Jyh-Jou Chen, Shih-Bin Su, Chih-Cheng Lai, Chien-Ming Chao, Chao-Jung Tsao, Shang-Hung Chen, Jhi-Joung Wang

**Affiliations:** 1 Department of General Surgery, Chi Mei Medical Center, Liouying 73657, Taiwan; chiuchongchi@yahoo.com.tw; 2 Department of General Surgery, Chi Mei Medical Center, Tainan 71004, Taiwan; 3 Department of Electrical Engineering, Southern Taiwan University of Science and Technology, Tainan 71005, Taiwan; 4 Department of Cancer Center, Chi Mei Medical Center, Liouying 73657, Taiwan; wenlilin2012@gmail.com; 5 Department of Healthcare Administration and Medical Informatics, Kaohsiung Medical University, Kaohsiung 80708, Taiwan; hshi@kmu.edu.tw; 6 Department of Business Management, National Sun Yat Sen University, Kaohsiung 80424, Taiwan; 7 Department of Medical Research, Kaohsiung Medical University Hospital, Kaohsiung 80708, Taiwan; 8 Department of Emergency Medicine, Chi-Mei Medical Center, Tainan 71004, Taiwan; chienchenghuang@yahoo.com.tw; 9 Department of Senior Services, Southern Taiwan University of Science and Technology, Tainan 71005, Taiwan; 10 Department of Gastroenterology and Hepatology, Chi Mei Medical Center, Liouying 73657, Taiwan; jjchen@mail.chimei.org.tw; 11 Department of Occupational Medicine, Chi Mei Medical Center, Liouying 73657, Taiwan; shihbin.su@msa.hinet.net; 12 Department of Occupational Medicine, Chi Mei Medical Center, Tainan 71004, Taiwan; 13 Department of Leisure, Recreation and Tourism Management, Southern Taiwan University of Science and Technology, Tainan 71005, Taiwan; 14 Department of Intensive Care Medicine, Chi Mei Medical Center, Liouying 73657, Taiwan; dtmed141@gmail.com (C.-C.L.); ccm870958@yahoo.com.tw (C.-M.C.); 15 Department of Oncology, Chi Mei Medical Center, Liouying 73657, Taiwan; m961193@mail.chimei.org.tw; 16 National Institute of Cancer Research, National Health Research Institutes, Tainan 70403, Taiwan; bryanchen@nhri.edu.tw; 17 Department of Medical Research, Chi Mei Medical Center, Tainan 71004, Taiwan; 18 AI Biomed Center, Southern Taiwan University of Science and Technology, Tainan 71005, Taiwan

**Keywords:** laparoscopic, open surgery, non-metastatic colorectal cancer, surgical complication, oncologic outcome, single surgeon experience

## Abstract

The oncologic merits of the laparoscopic technique for colorectal cancer surgery remain debatable. Eligible patients with non-metastatic colorectal cancer who were scheduled for an elective resection by one surgeon in a medical institution were randomized to either laparoscopic or open surgery. During this period, a total of 188 patients received laparoscopic surgery and the other 163 patients received the open approach. The primary endpoint was cancer-free five-year survival after operative treatment, and the secondary endpoint was the tumor recurrence incidence. Besides, surgical complications were also compared. There was no statistically significant difference between open and laparoscopic groups regarding the average number of lymph nodes dissected, ileus, anastomosis leakage, overall mortality rate, cancer recurrence rate, or cancer-free five-year survival. Even though performing a laparoscopic approach used a significantly longer operation time, this technique was more effective for colorectal cancer treatment in terms of shorter hospital stay and less blood loss. Meanwhile, fewer patients receiving the laparoscopic approach developed postoperative urinary tract infection, wound infection, or pneumonia, which reached statistical significance. For non-metastatic colorectal cancer patients, laparoscopic surgery resulted in better short-term outcomes, whether in several surgical complications and intra-operative blood loss. Though there was no significant statistical difference in terms of cancer-free five-year survival and tumor recurrence, it is strongly recommended that patients undergo laparoscopic surgery if not contraindicated.

## 1. Introduction

Since the first laparoscopic-assisted colon resection introduced in 1991 by Jacobs et al., it has gradually become popular [1]. Increasingly more colorectal surgeons admit that the laparoscopic technique leads to quicker functional recovery [2,3,4,5] and improved short-term results when compared with the open approach [6,7,8,9,10,11,12]. However, the laparoscopic technique has not previously been proven to gain significant benefits in colorectal surgeries [13,14,15,16,17]. Recently, oncologic outcomes of colorectal cancer resection, in terms of lymph node harvest number and excision safety margin lengths, achieved under laparoscopy could be comparable to those obtained using the conventional open technique. However, the curability of colorectal cancer under the laparoscopic technique remains controversial because of the uncertainty about the overall recurrence rate [18]. Besides, three principal, randomized clinical trials have proven that the laparoscopic technique can lead to the same oncological outcomes related to an open approach, but did not distinguish a survival benefit favoring laparoscopy [2,4,6].

It is believed that the role of the laparoscopic technique for advanced non-metastatic colorectal cancer management will be clarified through this study. The aim of this research was a comparison of surgical complications and five-year oncologic results of non-metastatic colorectal cancer patients receiving laparoscopic resection (LR) or open resection (OR) by one surgeon in a medical institution.

## 2. Materials and Methods

### 2.1. Ethics Statement

The institutional Ethics of Research Committee of Chi Mei Medical Center, Taiwan permitted this study. The protocol conformed to ethical standards according to the Declaration of Helsinki published in 1964. Moreover, written or verbal consent from patients was acquired for this study.

### 2.2. Study Population

**Figure 1:**
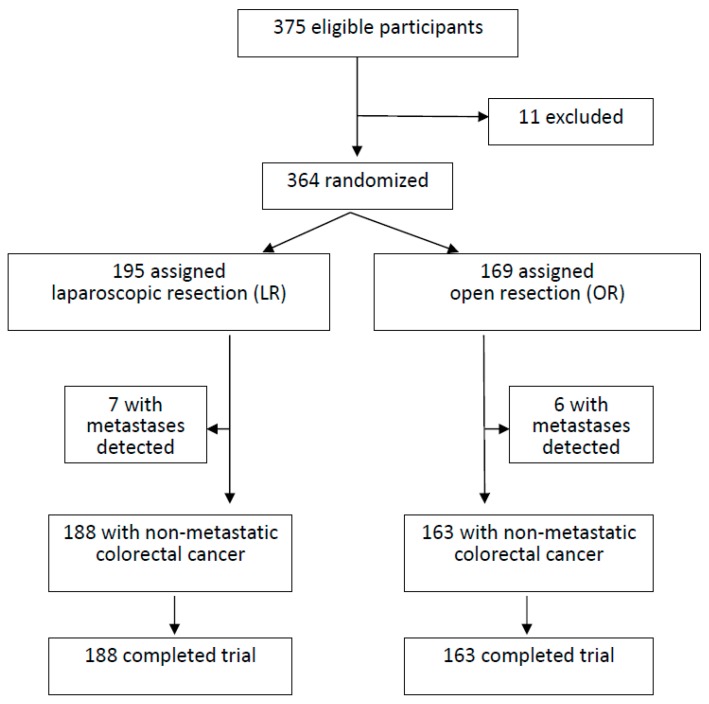
The flowchart of the study design.

From January 2008 to December 2013, a total of 375 consecutive colorectal cancer patients scheduled for resection by Dr. Chiu in a regional hospital with LR or OR were assessed (Figure 1). The treatment protocol was based on the National Comprehensive Cancer Network (NCCN) Guidelines^®^. The exclusion criteria included patients with cancer distant metastasis, synchronous tumors, adjacent organ invasion, intestinal obstruction, combined operations for other disease, history of trans-abdominal or trans-anal colorectal surgery, history of inflammatory bowel disease, polyposis, past episode of ileus related to severe intra-abdominal adhesions, morbid obesity, severe medical disease, pregnancy, emergent surgeries, patient unwilling to participate in the study, or conversion to open approach. Conversion to open approach was defined as an abdominal incision larger than necessary for specimen retrieval. Written informed consent was obtained from all patients in this study. This study divided patients into several groups according to the tumor locations. Patients of each tumor location group were randomly allocated to receive LR or OR using the random numbers belonging to that location group in the envelopes, blindly selected by the surgeon before the operation. In the LR group, all patients needed to pay for the extra fee of the harmonic scalpel and wound retractor. Data were collected in a prospectively maintained database that was supplemented by a retrospective chart review.

### 2.3. Pre-Operative Staging Work-Up

The evaluation included physical examination, colonoscopy with biopsy, abdominal, and pelvic computed tomography (CT) scan. Pelvic magnetic resonance imaging was routinely performed for rectal cancer patients. Serum level of carcinoembryonic antigen (CEA) was sampled before the operation. The pre-operative clinical oncologic staging was classified by tumor node metastasis (TNM) system of the American Joint Committee on Cancer (AJCC)/International Union Against Cancer (UICC).

### 2.4. Surgical Techniques

All LR and OR procedures proceeded with a standardized medial-to-lateral approach and non-touch technique. During LR surgery, the surgeon and camera operator stood on the opposite side of the colorectal lesion, while the first assistant positioned to the same side of the lesion. Briefly, the right hemicolectomy including the range extended to the mid-transverse colon with lymphadenectomy about the ileocolic, right colic, and middle colic vessel origin was selected for proximal lesions (those sited proximal to the flexure of the spleen). The left hemicolectomy with lymphadenectomy at the level of the left colic and the left branch of the middle colic vessel origins was selected for lesions at the descending colon. The omentum was transected to allow entry into the omental bursa and mobilization of the liver flexure (right hemicolectomy) or splenic flexure (left hemicolectomy). As lesions of the sigmoid colon or rectosigmoid junction, the sigmoid colectomy with upper rectum resection and lymphadenectomy extended to the inferior mesenteric vessel origin were selected. At least 5 cm safety surgical clearance margin was mandatory for all patients. As for rectal cancer, the technique was standardized as follows: (1) for upper third rectal lesions, a 5 cm mesorectal resection with end-to-end colorectal anastomosis was done; (2) for mid and low rectal lesions, total mesorectal excision with pouch supra-anal or anal anastomosis was performed; and (3) abdominoperineal excision was indicated once the levator muscle was involved by tumor. According to the principle of the non-touch technique, high ligation of the inferior mesenteric artery and mobilization of the splenic flexure were first systematically performed, whether the procedures were performed in LR or OR group. Dissected tissue was pulled out via a wound retractor at the extended umbilical wound for abdominal wall protection. For proximal lesions, anastomosis was routinely performed extra-corporeally. We routinely performed trans-anally intra-corporeal circular stapled anastomosis after the descending colon, sigmoid colon, or rectum lesion resection, because the residual distal intestine stump after resection was hardly managed extra-corporeally. In the OR group, the procedures were performed through a midline laparotomy with the same rules, and the wound was protected by gauze covering. The harmonic scalpel was generally used for soft tissue dissection in the LR group, but not in the OR group. Besides, in cases with difficult tumor localization by vision or palpation, an intra-operative colonoscopy would be routinely used to locate the actual tumor site instead of using other methods. However, no one in the OR group needed an intra-operative colonoscopy, but only three patients in the other group needed one.

### 2.5. Post-Operative Management

Post-operative treatment was the same for both groups. Patients were discharged when they had sufficient oral intake, well-controlled complications, or no complications. Complications designated as more severe than grade I according to the Clavien–Dindo classification system were categorized as ileus, urinary tract infection, wound infection, pneumonia, anastomotic leakage, and so on. Besides, most patients with stage III colorectal cancer would receive post-operative chemotherapy (oral or intravenous form), except six patients of the LR group and three of the OR group owing to general weakness or intolerance to chemotherapy.

### 2.6. Post-Operative Follow-Up

One specialized pathologist assessed all specimens. All patients were followed up with clinical examination, serum CEA assay, chest X-ray exam every three months, and liver ultrasound every six months for the first two years, and then annually. An abdominal CT exam was arranged annually. A colonoscopy was performed at one year after the operation, then every three years.

### 2.7. Statistical Analysis

**Figure 2:**
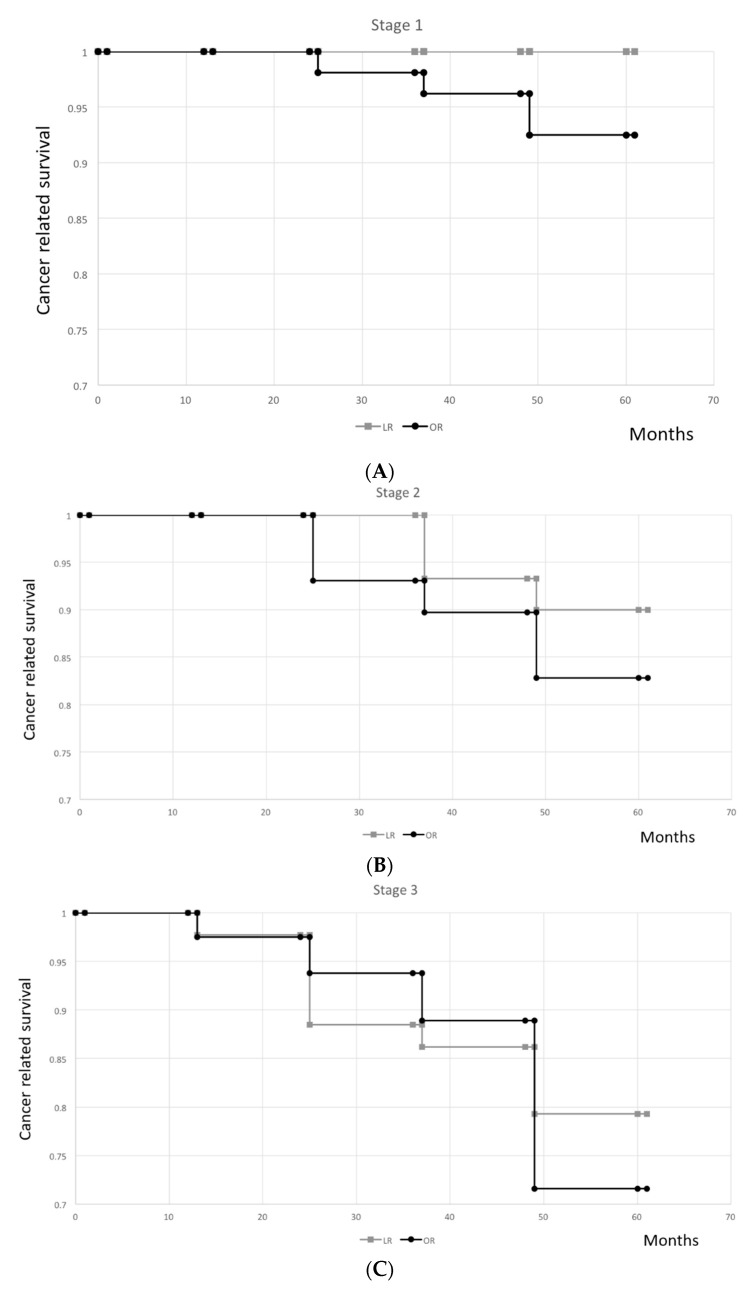

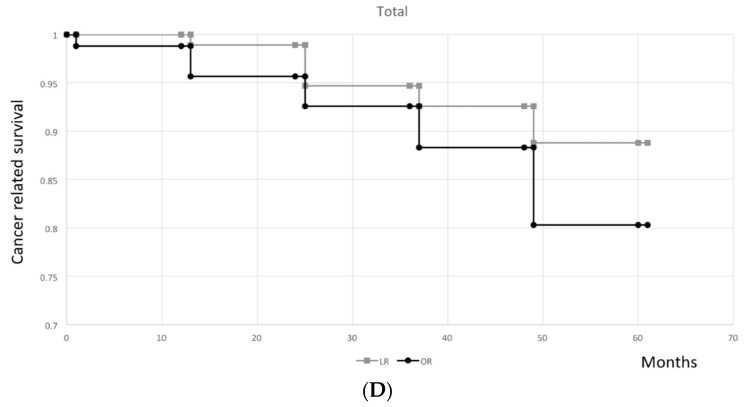
(**A**) Kaplan–Meier curve of cancer-free five-year survival in stage I patients (*p* = 0.206); (**B**) Kaplan–Meier curve of cancer-free five-year survival in stage II patients (*p* = 0.713); (**C**) Kaplan–Meier curve of cancer-free five-year survival in stage III patients (*p* = 0.426); (**D**) Kaplan–Meier curve of cancer-free five-year survival in all stage patients (*p* = 0.328).

The main endpoint of this study was cancer-free five-year survival. The secondary endpoint was the incidence of tumor recurrence. Predefined baseline variables are listed in Table 1 and Table 2. Variables for the univariate analysis were gender, age, American Society of Anesthesiologists (ASA) class, tumor location, TNM stage, histopathology, pre-surgery serum CEA level, type of intervention, postoperative complications, and tumor recurrence. Categorical variables were compared using the *χ*^2^ test. Continuous variables (e.g., number of lymph nodes removed, hospitalization period, intra-operative blood loss, and operation time) were compared using Student’s *t*-test. Survival period was evaluated from the day of surgery to the last visit or death. For cancer-free survival, patients dying from other causes were censored at the time of death. Probability curves were constructed according to the Kaplan–Meier method and compared with the log-rank test (Table 3) (Figure 2). A *p*-value less than 0.05 was regarded as statistically significant. All calculations were performed using the SPSS software package version 20 (SPSS Inc., Chicago, IL, USA).

## 3. Results

### 3.1. Baseline Characteristics of Patients

The basic profile of this study is shown in Figure 1. Initially, 375 colorectal cancer patients under Dr. Chiu’s service were sorted. Of these, 11 were excluded from the study. A total of 364 patients receiving curative resection were assessed in this study; 195 received LR and 169 received OR. Carcinomatosis was detected intra-operatively in seven patients of LR and six patients of OR, which were excluded. The remaining patients were compliant with the follow-up protocol. The median surveillance period was about 60 months.

In Table 1, both groups of patients were well matched in terms of demographic and clinicopathologic parameters. During this study period, 188 patients of the LR group were compared with the data obtained from the other 163 patients of the OR group. In the LR group, the mean age was 68.6 ± 12.7 years, and 102 (54.3%) patients were male. According to the final pathology report, three patients were classified in stage 0, 68 in stage I, 30 in stage II, and 87 in stage III. In the OR group, the mean age was 71.5 ± 12.1 years, and 87 (53.4%) patients were male; none were affected by tumors in stage 0, 53 in stage I, 29 in stage II, and 81 in stage III. There was a little disparity between clinical/radiologic staging and pathological staging in this study. Other characteristics of tumors and patients were summarized, and there was no statistical difference between these two groups.

### 3.2. Surgical Outcomes

In Table 2, the rate of tumor recurrence was 9.0% (17/188) in the LR group and 13.5% (22/163) in the OR group. Although the difference was not statistically significant, tumor recurrence seemed to be lower in the LR group (*p* = 0.186). The average number of lymph nodes removed in LR was 16.0 ± 9.2 and 19.2 ± 13.7 in OR (*p* = 0.07). Tumor margins were non-involved in patients of both groups. However, this study demonstrated that LR was more effective for the treatment of colorectal cancer in terms of hospital stay (*p* < 0.001) and blood loss (*p* < 0.001). Conversely, operation time was significantly longer in LR than in OR (191.4 ± 71.1 min vs. 150.8 ± 46.3 min, *p* < 0.001). Compared with the LR group, more patients in the OR group encountered postoperative urinary tract infection, wound infection, and pneumonia, which reached statistical significance. Only two patients in the OR group were found with mild anastomosis leakage from the drainage tube clinically. Abdominal CT confirmed the diagnosis and that the degree was mild. These two patients received conservative treatment, including intravenous fluid supply and nil per os. However, further surgical intervention was not necessary.

### 3.3. Cancer-Free Survival Rates and Tumor Recurrence Incidence

In Table 3, twenty-one patients (11.2%) of the LR group and 32 patients (19.6%) of the OR group expired. There was a trend of higher overall mortality in the OR group, with 4 in stage I, 5 in stage II, and 23 in stage III, but it was not statistically different. In stage 0, there were only three patients in the LR group and none in the other. All three patients survived more than five years after surgery. In stage I, all four deaths of the OR group were non-cancer related. All patients survived for at least 30 months after surgery. In stage II, two patients in the OR group died within the second year after surgery, but they were non-cancer related. Others in both groups who died were all cancer-related. In stage III patients, all eighteen deaths in the LR group and twenty-three deaths in the OR group were cancer-related. In the OR group, two patients died fewer than six months after a second oncologic surgery for cancer recurrence, about three years after previous surgery.

There was a phenomenon of a higher cancer-free five-year survival in stage I (*p* = 0.206, Figure 2A), stage II (*p* = 0.713, Figure 2B), stage III (*p* = 0.426, Figure 2C), and all stages (*p* = 0.328, Figure 2D) in the LR group when compared with those in the OR group, although the difference was not statistically significant.

**Figure 3:**
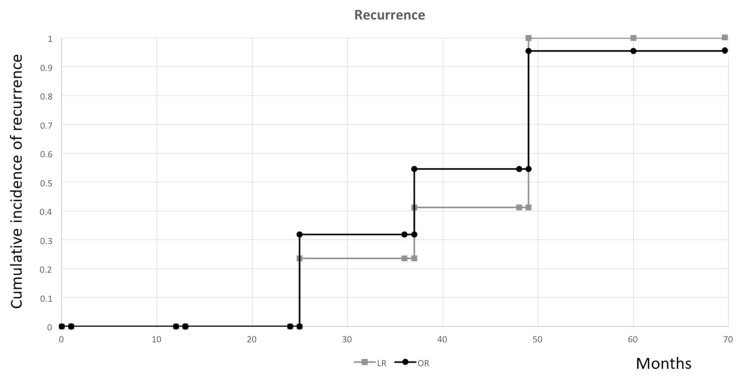
Cumulative incidence curve of tumor recurrence in all stage patients (*p* = 0.186).

The median time for tumor recurrence was 57.0 months (range 25–68 months) in LR and 53.5 months (range 25–63 months) in OR. Importantly, no difference was observed in the cumulative incidence of recurrence between these two groups (*p* = 0.186) (Figure 3). Besides, there was no incidence of port-site recurrence in the LR group or wound recurrence in the OR group.

## 4. Discussion

Previously, randomized controlled studies demonstrated that LR had favorable operative outcomes with less wound pain, earlier functional return of the gastrointestinal tract, a shorter hospital stay, and better cosmetics when compared with OR [19,20,21,22]. Moreover, a meta-analysis [23] and two large retrospective studies [24,25], which included a large number of patients, also showed a significant reduction in the mortality rate and lowered the morbidity after LR.

However, survival is the most crucial concern for assessing success for malignant disease treatment. This study included a 60-month follow-up and compared LR and OR for non-metastatic colorectal cancer. The results of cancer-related survival and incidence of tumor recurrence favored the LR group, despite that there was no statistically significant difference regarding the oncological results. The Clinical Outcome of Surgical Therapy study, which was the largest randomized controlled trial conducted so far, also showed the same results as ours and even overall survival between the two groups after a median four-year follow-up [2]. However, in a single institution randomized study, Lacy et al. advocated that there was a cancer-related survival advantage after LR for stage III colon cancer patients [26]. Capussotti et al. also demonstrated that LR was related to significantly better disease-free and cancer-related survival stage III colon cancer patients [27]. Other studies have reported better survival for patients undergoing LR, even for those with stage II colorectal cancer [28].

One of the assumptions about better survival might be the number difference in dissected lymph nodes between the LR and OR groups. Laparoscopy provides better visualization of intra-abdominal conditions [29], including a more comprehensive, more precise, and brighter image to allow surgeons to perform a more radical and precise resection of the mesocolon and mesorectum, while facilitating an accurate and complete lymphadenectomy [30,31]. Complete lymphadenectomy for colorectal cancer is essential for the patient’s oncological prognosis because of a reduced risk of residual nodal disease, as well as accurate nodal staging (achieving a better stratification of tumor staging) [19]. However, there was no statistical difference in the lymph node retrieval number between these two groups. Similarly, the retrieved and assessed lymph node number in many patients of both groups was higher than the threshold of 12 lymph nodes recommended by the American Joint Committee on Cancer (AJCC) in our study. Lymphadenectomy of colorectal cancer was a decisive factor for the prognostic and therapeutic staging of the patient. Different variables could affect the retrieval number of lymph nodes. Some, like the surgeon, the surgery, and the pathology exam, were without question modifiable; however, other both patient- and disease-related variables were non-modifiable and posed the question of whether the minimum number of examined lymph nodes must be individually assigned. However, since 2010, the AJCC classification subdivided patients treated for colorectal cancer into prognostic categories according to the number of metastatic lymph nodes [32]. The accuracy of the staging was influenced by the number of retrieved lymph nodes as the relationship between positive nodes divided by the total number of retrieved nodes. With regard to the prognosis prediction, this “lymph nodal ratio” is also effective in cases of reduced lymph nodal sampling [33,34,35,36]. Besides, the sentinel lymph nodes were thought to find valid application in this field in the future [37]. Of course, improvement of these modifiable factors is the only aspect that our team could strive for at this moment, as well as the opportunity to gain a better oncologic outcome of cancer remission or recurrence after reducing the risk of residual nodal disease.

Other proven benefits in oncologic results about LR include its effect on cellular immunity, intra-operative tumor manipulation, related stress response and subsequent cytokine release, surgical complication rate, and blood transfusion amount [26]. Conclusively, one of the most essentially beneficial theories of LR is regarded as the preservation of the patient’s immunological response against cancer from the first postoperative days [38]. There has been significant evidence suggesting that surgical stress interferes with immunity, and this phenomenon is more apparent in OR than in LR [39]. The role of immunosuppression has been advocated because immunologic response mediators (e.g., C-reactive protein, interleukin 1–6, and tumor necrosis factor alpha) are decreased after LR in colorectal surgery compared with the OR approach. On the other hand, immunosuppression deteriorates both sepsis and cancer cell proliferation [40]. Lacy et al. have also pointed out that the post-LR stress response of colorectal cancer is less pronounced and finally leads to better preservation of cellular immune function, and attenuates inflammatory mediator interference [41,42]. Correlation of the stress response degree after the trauma of surgery with the host resistance to cancer has been proven in an animal model [26]. Immunity is a critical barrier against tumor progression and metastatic spread [39]. LR could, therefore, theoretically increase either overall or cancer-free survival. However, we should routinely examine these immunologic response mediators after surgery to improve the quality of our further study.

Tumor manipulation has been proven to contribute to cancer cell spread. There is some evidence that tumor mobilization is related to cancer cells’ exfoliation into the peritoneal cavity and portal vein bloodstream migration, which might be alleviated by non-touch surgical techniques or the avoidance of tumor manipulation. Preliminary reports have shown that cancer cell spread is not worsened [43], and dissemination of cancer cells is reduced by LR [39]. However, this phenomenon is difficult evaluated in this study based on the safety issue of blood sampling from portal vein bloodstream. Under the laparoscopic vision, limited access inside the abdominal cavity leads to minimal tumor handling and compliance of non-touch technique, both favoring the important oncology principle to avoid tumor cell spread during surgery. In this study, all patients received non-touch isolation techniques in both the LR and OR groups, which should cause no difference in prognosis. In the future, we could also perform intra-operative abdominal cavity normal saline irrigation after tumor resection to compare the difference of possible exfoliated cancer cells into the peritoneal cavity by two surgical techniques.

There is an evident statistical difference in fewer complication rates and the amount of blood loss in the LR group compared with the OR group. These factors theoretically contribute to better prognosis of tumor recurrence and cancer-free survival in LR patients. Despite that the differences regarding the oncological results did not reach the statistical significance of both groups, other better short-term outcomes, including smaller incisions, less postoperative pain, quicker functional recovery, shorter hospital stays, and earlier return to regular activity, suggested colorectal cancer patients should receive LR if not contraindicated. Meanwhile, although the operation length was longer in our LR group, the benefit of this minimally invasive technique on peri-operative care (quicker functional recovery and shorter hospital stays) further overcame this disadvantage. Besides, this benefit in shorter peri-operative care would significantly lower the total medical cost, especially in Western countries. Meanwhile, it is believed that we could set up some standard protocols and guidelines for routine use to decrease the operation time in the OR group in the future.

There are many debates about the effect of wound size. Several experienced colorectal surgeons pointed out that most colectomies could be performed with an abdominal wound of less than 7 cm, and thus opposed the wound benefits of LR [44]. However, the advantages of LR for colorectal cancer not only include a comparatively smaller wound size, but also relate to the properties of laparoscopy, especially the operation field magnification, more precise tumor resection, and its minimal invasiveness [5]. One meta-analysis including 3863 patients even showed that single-incision laparoscopic surgery (SILS) had comparable outcomes to LR in terms of operating time, conversion rate, reoperations, postoperative complications, and mortality, but only shorter mean hospital stay. There was no difference in the oncological results regarding average lymph node retrieval, adequate resection margins, survival rates, and local recurrence [45].

Application of LR for colorectal cancer encountered much criticism in the early 1990s as a result of several case reports about port site recurrence and suspicion of the adverse effect of oncologic outcome [25]. However, many surgeons advocated that LR did not aggravate cancer cell spillage intra-corporeally when surgeons strictly followed the oncologic principles [5]. However, the routine practice of the laparoscopic technique in colorectal cancer treatment is only performed in a few experienced centers in Taiwan. Localization of the tumors remains a major limiting factor of LR popularity among most surgeons, despite that there are several techniques, including conventional colonoscopy and colonoscopic tattooing, colonoscopic clip placement, radio-guided colorectal lesion localization, and the application of magnetic colonoscopic imaging [46]. Besides, some specialists are still quite hesitant about laparoscopy because of the lack of “direct” physical and visual contact of the lesion. Particularly during the LR process, the intestinal color is more difficult to assess, and direct palpation of blood vessels to the anastomosis is not possible [47]. The phenomenon of the slow popularity of this minimally invasive technique further reflects its complexity, especially at the initial stage of the learning curve; the lack of three-dimensional visualization, the absence of safe laparoscopic instruments, and the paucity of tactile feedback are still usually the causes of barriers to popularity and the causes of conversion during surgery [48].

Moreover, practicing a new or pioneer surgical technique on patients with a malignant disease is not permitted in the ethical aspect. However, inreasingly more improved new techniques of LR are being explored [47]. For the majority of cases, pioneers feel confident to employ LR. If further efforts are made to achieve standardization of these minimally invasive procedures and improvement of the related educational system, LR will undoubtedly become the standard and mainstay therapy for many bowel diseases, besides colorectal cancer. Furthermore, it is expected that other new techniques such as reduced port surgery and robotic surgery will be confirmed efficient and safe in the future [49].

Many experts pointed out that the learning curve for laparoscopic colorectal surgery is about greater than twenty cases [50]. In 2013, one meta-analysis by Comité de l’évolution des pratiques en oncologie (CEPO) recommended that LR be considered an option for the curative treatment of colon and rectal cancer in consideration of surgeon experience, tumor stage, potential contraindications, and patient expectations. Instead, CEPO also suggested only competent experts with sufficient annual surgeon volume should perform LR for rectal cancer patients [51]. However, safety control, quality monitor, and technique standardization applied to the surgical aspects of the study would provide a solution to the learning curve issues by the collaboration of interested experts to set up safe and reproducible experiment steps even in the setting of new technology [52]. As for the surgeon on our team, he had experience with LR of more than 100 cases before this study. Thus, the learning curve effect of our study series was not discussed.

Compared with previously published randomized studies in the literature, there were some weak points of this study that needed to be further addressed. Our hospital was an 800-bed regional hospital with a total of around 120 colorectal cancer operations per year. Admittedly, the number of patients included in this study was too small (only 351 patients) for comparison of the oncological outcomes; we should increase the sample size to make a reliable comparison between these two groups and to avoid the related bias in the future. Second, our surgeon excluded morbidly obese patients in this study because our surgeon preferred them to receive LR to lower the incidence of possible abdominal wound herniation in the future, which might cause a potential bias. However, only one morbidly obese patient was encountered during the study period, and he was excluded because he encountered the problem of severe intestinal adhesion and received conversion from LR to the OR approach. Third, recently developed and popular trans-anal surgery for patients with rectal lesions was not discussed in this study because we could not compare traditional OR and LR techniques. Fourth, some patients might encounter the problem of their retrieved and assessed lymph node number being lower than the threshold of twelve, which might cause inaccurate TNM staging and select inappropriate minor treatment in their protocol.

In Table 2, the result of our anastomosis leakage rate (0.57%) compared favorably to those of the published literature (0.9%–3.5%). Higher leak rates were typically reported for low pelvic anastomoses or anastomoses to the anal canal [53]. There were three reasons for this comparatively “better” result in our study. First, we largely selected surgical patients with good pre-operative nutrition status (Subjective Global Assessment of Nutritional Status class A or B). Second, we preferred to perform protective diversional stoma for patients with a higher risk of anastomosis leakage, especially those having pre-operative concurrent chemoradiotherapy. We believed nearly all small contained leaks of the anastomosis site would heal after fecal diversion for about six months. Third, the true incidence of anastomosis leakage was underestimated. We only performed post-operative CT for patients when turbid or stool-like discharge was noted from the drainage tube near the anastomosis site. Pickleman et al. advocated that some colorectal surgical patients ultimately found to have an anastomosis leakage developed a more insidious presentation, often with low-grade fever, prolonged ileus, or failure to thrive [54]. In these patients, confirmation of the diagnosis might be much more difficult, as the clinical course was often similar to other postoperative infectious complications. Radiologic imaging was usually required; even then, the definitive diagnosis might be elusive or at least uncertain [53]. Although there have been many studies that specify a rate of anastomotic leakage, it is seldom possible to know what constitutes a “leak”. Bruce et al. performed a systematic review of studies measuring the incidence of anastomotic leaks after gastrointestinal surgery; in the 97 studies reviewed, there was a total of 56 separate definitions of the anastomotic leak [55]. A leak may be defined by the need for reoperation, clinical findings, or radiologic criteria (CT scanning or contrast enema), making “accurate” comparisons between these studies difficult or impossible [53].

Meanwhile, colorectal cancers at different sites were included for analysis of oncologic outcomes and functional results in our study. This study design was debatable because the lymphatic drainage route, range of dissection during tumor resection, operation techniques, and even the biologic behavior were different in various colorectal locations [5]. However, all patients of the LR and OR groups were treated by a single surgeon, and this could avoid the related bias when patients were treated by multiple surgeons. Besides, if we could increase the patient number (sample size) in the future, we could analyze and discuss the tumor located at one specific site to decrease this bias.

Patients with liver metastasis (a common metastasis area of colorectal cancer) were excluded in our study because we thought these terminal patients (defined by current TNM staging system) should receive systemic treatment if no evident clinical lumen obstruction. However, in China, the attitudes of therapeutic approaches in these patients seem to vary among areas [56]. Specific guidelines regarding liver metastasis were revised in 2018 in order to improve the diagnosis and treatment strategy, including the overall clinical evaluation, personalized treatment goals, and comprehensive treatment protocol, in order to prevent the occurrence of liver metastases, and improve the resection rate of liver metastases and survival [57]. Although experts of different countries have investigated their treatment strategy for colorectal cancer thoroughly for many years, the TNM staging system is still commonly regarded as an essential tool to predict oncologic outcomes [58]. It has made an essential contribution to the clinical management of cancer patients over the past 50 years [59], but are we sure it delivers what is needed to provide adequate advice in the 21st century? Would patients face different oncologic outcomes despite that they have been labeled with the same TNM stage clinically?

Nowadays, the degree of cancer infiltration, the number of lymph nodes involved, and distant metastasis have generally been accepted as the most paramount items to predict outcomes [60]. Nevertheless, some patients in the same clinicopathological stage might exhibit unique variation in outcomes with different rates of cancer recurrence and mortality when merely evaluated with the current TNM staging system [60,61]. Specialists conducted intensive studies about the possible causes of this discrepancy. Maguire et al. pointed out that the classification of peritoneal involvement was different in TNM 5 and TNM 7. The Royal College of Pathologists in the United Kingdom still recommended the use of the TNM 5 staging system, while TNM 7 had been adopted in many other jurisdictions. In TNM 5, a tumor directly invading other organs was staged as pT4a, while a tumor involving the visceral peritoneum was staged as pT4b [60]. However, the reported incidence of peritoneal involvement ranged from 5% to 43% in studies of stage II colorectal cancer, which led to a wide statistical variation and an unreliable result [62,63,64,65,66,67,68,69]. Besides, Puppa et al. also advocated that identification and classification of morphologic features encountered in the pathologic examination of colorectal cancer specimens might be difficult and a source of subjective variability. They suggested that enhanced pathologic analysis, agreed-upon standard protocols, and standardization should improve the completeness and accuracy of pathology reports. In other words, the optimal staging system of colorectal cancer should encompass both anatomic and nonanatomic factors, the latter including molecular and treatment factors [61]. Some oncologists also advocated that cancer development and progression might depend partly on “changes” in several histological features, which might lead to this discrepancy clinically. These previously unrecognized features were closely related to the way cancerous cells interact with the surrounding stroma and obtain their potential for invasiveness [70]. These characteristics included tumor budding, poorly differentiated clusters, extramural vascular (vein) invasion, perineural invasion, tumor deposits, and mucin pools [58]. This discrepancy in the molecular signature of colorectal cancer has also revealed differences in phenotypic aggressiveness and therapeutic response rates [58]. Thus, we should remind colorectal cancer patients of the potential risk of having a disappointing result when choosing inappropriate minor post-operative treatment. The discrepancy in staging colorectal cancer has critical effects on management, outcomes, and survival rates of the patients. Accurate predictions of the final pathological disease stage using high quality, accurate pre-operative clinical-radiological staging techniques enables multidisciplinary teams to plan prompt optimal management strategies for patients with colorectal neoplasms [71]. As cancer clinicians strive to improve survival by increasingly smaller steps, the accuracy of TNM staging becomes even more critical in the interpretation of reports of further clinical trials [59]. More importantly, it is essential to introduce effective preventive measures to this increasing global disease [72].

## 5. Conclusions

Within the limitations of this study, the results showed better short-term outcomes in terms of postoperative urinary tract infection, wound infection, pneumonia, and blood loss in LR versus OR for non-metastatic colorectal cancer. Although the differences regarding cancer-free five-year survival and tumor recurrence did not reach the statistical significance of both groups, it is strongly recommended that patients undergo laparoscopic surgery if not contraindicated.

## Figures and Tables

**Table 1:** Comparison of baseline characteristics between laparoscopic resection (LR) versus open resection (OR).

Items	LR (*n* = 188)	OR (*n* = 163)	*p*-Value
Gender			0.435
Male	102	87	
Female	86	76	
Age (mean ± SD)	68.6 ± 12.7	71.5 ± 12.1	0.23
ASA class			0.698
I	113	92	
II	75	71	
TNM stage (clinical/radiologic)			0.345
0	3	0	
I	70	55	
II	26	26	
III	89	82	
TNM stage (pathologic)			0.344
0	3	0	
I	68	53	
II	30	29	
III	87	81	
Histopathology			0.624
Well differentiated	92	79	
Moderate differentiated	71	65	
Poorly differentiated	25	19	
Tumor location			0.431
Cecum	29	20	
Ascending colon	41	36	
Transverse colon	12	15	
Descending colon	21	18	
Sigmoid colon	52	45	
Rectum	33	29	
Intervention			0.720
Right hemicolectomy	65	56	
Left hemicolectomy	24	21	
Transverse colectomy	14	10	
Sigmoid colectomy	50	44	
Protectomy	32	27	
Abdominal perineal resection	3	5	
Protective diversional stoma	13	15	
Pre-surgery			1.000
serum CEA level			
<5	24	13	
≥5	164	150	

ASA—American Society of Anesthesiologists; TNM—tumor node metastasis; CEA—carcinoembryonic antigen.

**Table 2:** Comparison of surgical outcomes between laparoscopic resection (LR) versus open resection (OR).

Items	LR (*n* = 188)	OR (*n* = 163)	*p*-Value
Tumor recurrence	17 (9.0%)	22 (13.5%)	0.186
Lymph nodes removed	16.0 ± 9.2	19.2 ± 13.7	0.07
Hospitalization (days)	13.2 ± 4.2	18.8 ± 9	<0.001 **
Blood loss (mL)	23.5 ± 14.6	162.2 ± 63.4	<0.001 **
Operation time (min)	191.4 ± 71.1	150.8 ± 46.3	<0.001 **
Postoperative complications			
Total	8	25	
Ileus	3	5	0.273
Urinary tract infection	1	5	<0.001 **
Wound infection	2	7	<0.001 **
Pneumonia	2	6	0.048 *
Anastomosis leakage	0	2	0.140

* *p* ≤ 0.05 ** *p* ≤ 0.001. LR Tumor recurrence: liver metastasis ×13, lung metastasis ×5, carcinomatosis ×4, anastomotic recurrence ×1, local recurrence ×3; OR Tumor recurrence: liver metastasis ×19, lung metastasis ×8, carcinomatosis ×6, anastomotic recurrence ×3, local recurrence ×2.

**Table 3:** Cancer-free survival rates between laparoscopic resection (LR) versus open resection (OR).

Stage	Group	*n*	Death		Survival					
					1st Year	2nd Year	3rd Year	4th Year	5th Year	*p*-Value
			*n*	%	% (*n*)	% (*n*)	% (*n*)	% (*n*)	% (*n*)	
0	LR	3	0	0	100 (3)	100 (3)	100 (3)	100 (3)	100 (3)	-
	OR	0	-	-	-	-	-	-	-	
I	LR	68	0	0	100 (68)	100 (68)	100 (68)	100 (68)	100 (68)	0.206
	OR	53	4	7.5	100 (53)	100 (53)	98.1 (52)	96.2 (51)	92.5 (49)	
II	LR	30	3	10.0	100 (30)	100 (30)	100 (30)	93.3 (28)	90.0 (27)	0.713
	OR	29	5	17.2	100 (29)	93.1 (27)	93.1 (27)	89.7 (26)	82.8 (24)	
III	LR	87	18	20.7	100 (87)	97.7 (85)	88.5 (77)	86.2 (75)	79.3 (69)	0.426
	OR	81	23	28.4	97.5 (79)	93.8 (76)	88.9 (72)	82.7 (67)	71.6 (58)	
Total	LR	188	21	11.2	100 (188)	98.9 (186)	94.7 (178)	92.6 (174)	88.8 (167)	0.328
	OR	163	32	19.6	98.8 (161)	95.7 (156)	92.6 (151)	88.3 (144)	80.3 (131)	
